# Supplementary material for: Identification of miR-671-5p and Its Related Pathways as General Mechanisms of Both Form-Deprivation and Lens-Induced Myopia in Mice
Source: Curr Issues Mol Biol. 2023 Mar 2;45(3):2060–72. doi: 10.3390/cimb45030132 (PMC10047131; doi:10.3390/cimb45030132)
Supplement: Supplementary file 1 [file cimb-45-00132-s001.zip › cimb-2198248-supplementary.pdf]

Supplementary material

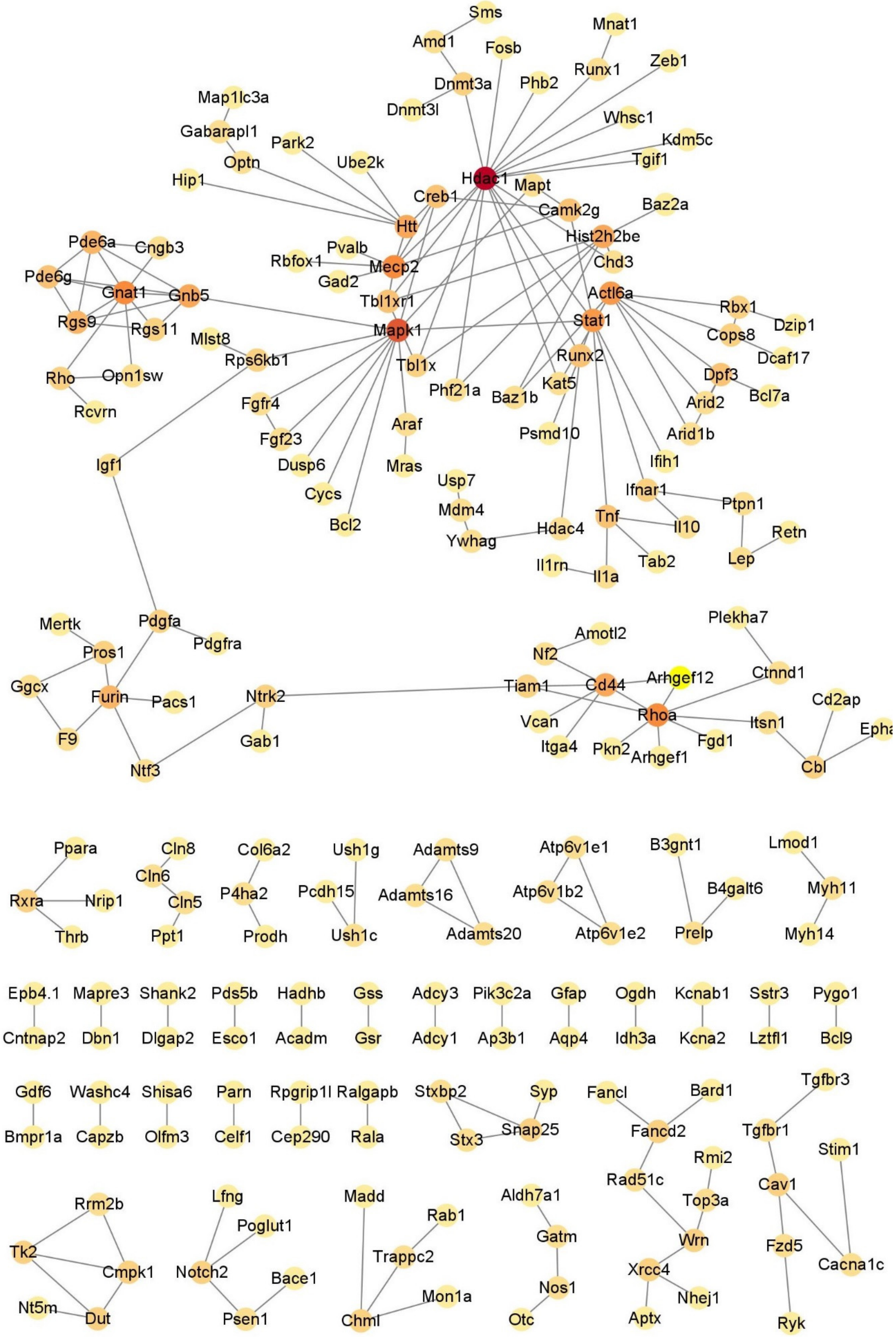

Supplementary Figure S1

The overview of the protein-protein interaction (PPI) network analysis of the overlapping genes. The 584 genes were input into the STRING database, and achieved a PPI network of 214 nodes and 428 edges. The nodes represent the query proteins, and the edges represent protein-protein associations. The color of the nodes was proportional to the number of degrees. The darker the node's color was, the more multi-functional the protein was.
